# Training spatial intelligence in football through the cognitive load scale

**DOI:** 10.3389/fspor.2025.1628561

**Published:** 2025-08-11

**Authors:** Rafael Pedrosa, Ricardo Tavares

**Affiliations:** ^1^ Neuroscience, Physiology and Pharmacology, University College London, London, United Kingdom; ^2^ Football Cognition, London, United Kingdom; ^3^ Federação Portuguesa de Futebol, Lisbon, Portugal

**Keywords:** spatial cognition, football (soccer), player development, cognitive scale, working memory, football practice policy, drill & practice

## Abstract

Cognitive skills like working memory and spatial awareness play a central role in football, yet they are not commonly addressed in training. Here, we propose a practical approach to integrating cognitive demands into football practice without losing the ecological validity of the game. We introduce the Cognitive Load Scale (CLS), a five-level framework to classify and adapt drills based on their cognitive demands. Through task constraints involving space, color rules, attentional shifts, and memory load, coaches can challenge how players perceive, decide, and act under pressure. We present examples across CLS levels, showing how spatial intelligence can be trained systematically on the pitch. The goal is to design sessions where the game itself becomes the tool for cognitive development.

## Introduction

Football performance requires the rapid integration of spatial and temporal information under time constraints. Players must follow the position of teammates and opponents, anticipate trajectories, and make quick decisions while performing complex motor actions. These demands heavily rely on cognitive resources such as working memory, attention, and spatial reasoning (1, 2).

Evidence from cognitive neuroscience suggests that brain systems involved in spatial information and decision-making are highly plastic and sensitive to task demands. Regions such as the hippocampus and entorhinal cortex support spatial mapping and route simulation (3, 4), and their activity patterns reorganize with experience and exposure to tasks-relevant constraints (5–7). This suggests that spatial intelligence can develop through experience and interaction with task-specific demands, particularly when training environments require continuous spatial updating and flexible decision-making. In sport contexts, this opens the door to designing training environments that engage and strengthen these systems directly (2, 8, 9).

In this article, we introduce the Cognitive Load Scale (CLS), a five-level framework for designing and adapting football drills based on cognitive demand. This framework allows coaches to implement cognitive aspects into training in a structured and scalable way, while preserving the dynamics of the game. It is designed to support the development of spatial intelligence in the pitch by creating conditions that challenge perception, memory, and decision-making under realistic constraints.

## Spatial intelligence in football

Spatial intelligence refers to the ability to perceive space, track movement, and respond to changes in the environment. In football, this means knowing where you are on the pitch, where others are, and how to move based on the structure of the game as it unfolds. These skills are crucial, especially under pressure, when quick and adaptive decisions rely on accurate spatial awareness.

From a neuroscience perspective, spatial intelligence involves brain areas such as the hippocampus, the entorhinal cortex, and parts of the parietal and prefrontal cortices. Research in rodents and humans has identified specific neurons involved in mapping space (4, 10–13):

• **Place cells** (hippocampus): fire when an individual is in a specific location in the environment.
• **Grid cells** (entorhinal cortex): activate in a hexagonal pattern that tiles space as the individual moves.
• **Head direction cells** (entorhinal cortex): encode which direction the head is facing, independent of location.
• **Goal-direction cells** (hippocampus): signal the direction or proximity of a target or goal location.

Together, these cells form what is often called a cognitive map, which helps individuals localize themselves and others, plan movements, and simulate future positions in space (Supplementary Figure S1).

This navigation system is closely linked to motor planning and memory. In football, players must continuously update spatial information as they move, tracking teammates, opponents, and the structure of play. The hippocampus and entorhinal cortex contribute to these spatial computations by estimating trajectories and maintaining internal representations of possible paths and targets (3). The prefrontal cortex interacts with these systems to support decisions that reflect both the context and the player’s goals (14, 15). Instead of relying only on external cues, players use internal models of space to guide their behavior on the pitch.

These spatial representations are also flexible. Experiments have shown that hippocampal activity reorganizes with experience. For example, place cells can shift or refine their firing as animals learn new environments or task structures (6, 7). In football, repeated exposure to structured spatial challenges, such as tracking open space, or holding spatial information in working memory, may similarly influence hippocampal coding. Over time, this process helps embed relevant spatial patterns into the athlete’s internal model of the game.

## Designing cognitive demand in football training

Spatial intelligence in football relies on the ability to read spatial information and update decisions based on the movement of others. These skills can be developed when training tasks consistently place players in situations where perception and decision-making are under pressure.

Most football sessions have focused on physical, technical, and tactical demands. Cognitive processes tend to emerge passively as the game becomes more complex. However, research suggests that they can be addressed more deliberately. The principle of desirable difficulty proposes that learning improves when players are challenged to solve problems, not just repeat actions (16, 17). Training can shift toward creating tasks that place players in uncomfortable cognitive spaces, where they must adjust, reflect, and reorganize their behavior.

Working memory has strict limits (18). When training pushes players close to these limits, they need to process information differently, often relying on more efficient internal strategies. Over time, this may help reinforce the cognitive systems that support flexible attention, prediction, and executive control. If designed carefully, these demands can be added without disrupting the rhythm or realism of the session.

## Cognitive priorities in transitions

Another natural source of cognitive overload in football arises from the shifting attentional hierarchies players must manage during transitions in play. When in possession, players tend to prioritize attention in the following order: (1) the ball, (2) teammates, and (3) opponents. Out of possession, this hierarchy typically shifts to (1) the ball, (2) opponents, and (3) teammates. These shifts require rapid reallocation of cognitive resources and continuous updating of internal representations as the context of play changes. During transitional moments, such as immediately after losing or regaining possession, players must quickly reverse their attentional priorities. This task-switching under pressure places high demands on working memory and executive functions, particularly cognitive flexibility and inhibitory control. Incorporating drills that emphasize or simulate these rapid attentional shifts can enhance players’ ability to adapt in real-time, offering a powerful form of game-relevant cognitive training.

## Training spatial intelligence in football

Football players operate under constant spatial and cognitive demands, often in conditions of high uncertainty and limited time. Traditional drills tend to focus on motor execution and tactical positioning, but rarely impose structured cognitive constraints. Here, we propose a set of training strategies designed to systematically engage spatial intelligence and working memory, while preserving the ecological validity of on-pitch scenarios.

**Figure 1:**
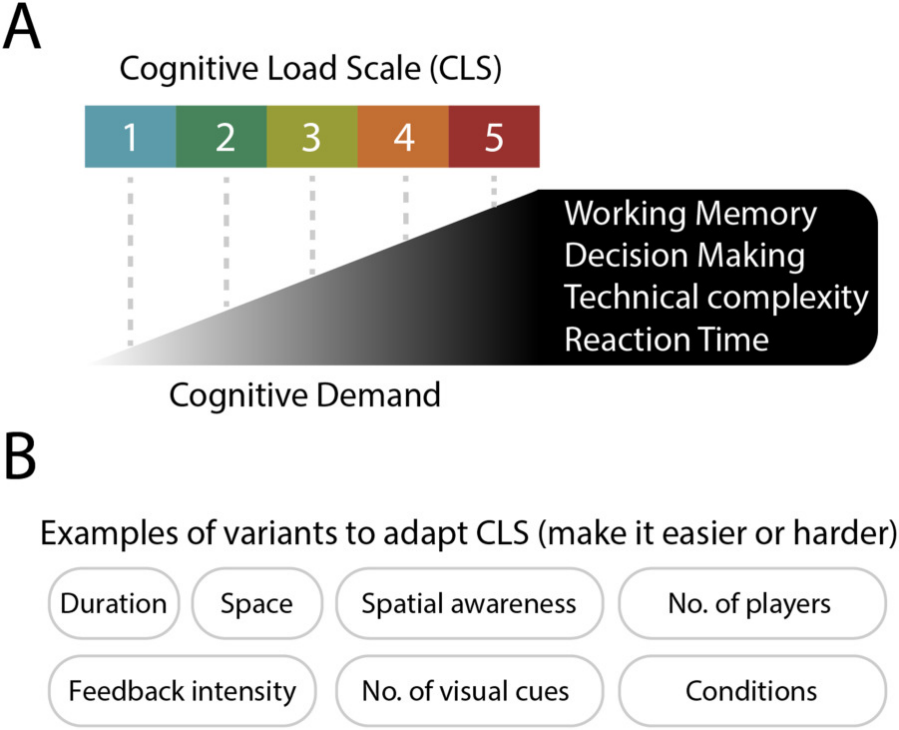
**(A)** Cognitive Load Scale (CLS) illustrating five levels of cognitive demand based on working memory, decision making, technical complexity, and reaction time. **(B)** Examples of adjustable task variables are shown, which can be used to increase or decrease the cognitive load during football training.

To support this, we introduce the Cognitive Load Scale (CLS), a five-level framework that captures the increasing cognitive demand placed on players across four key dimensions: working memory, decision making, technical complexity, and reaction time (Figure 1A). The CLS provides coaches with a practical way to design and adapt drills using modifiable variables such as space, duration, number of players, spatial awareness, feedback intensity, conditions, and visual cues (Figure 1B).

Table 1 provides a summary of the five CLS levels and how they differ across these core components, offering a practical guide to adjust task difficulty without losing the realism of the game.

**Table 1:** Comparative summary of the five CLS levels and their core cognitive components to support training design.

CLS level	Perception	Memory	Decision-making	Task characteristics
1–Minimal	Sees basic cues in a slow and clear setting	Relies only on habits or routines	Quick, almost automatic choices	Simple drills, familiar rules, lots of time and space
2–Low	Needs to notice and react to one new cue	Remembers a specific rule or cue added to the task	Chooses based on one added element	Slightly faster drills, one extra instruction, mild pressure
3–Moderate	Tracks several moving things at once	Keeps track of roles or simple changes	Makes decisions while adapting to the play	Game-like situations with changing roles or rules
4–High	Picks up subtle cues under pressure	Remembers short sequences and avoids habits	Needs to think ahead and adjust quickly	One-touch rules, pattern changes, limited time and space
5–Extreme	Switches attention quickly between multiple cues and tasks	Handles multiple rules and switches between tasks	Makes fast decisions in chaotic situations	Dual tasks, small space, high tempo, constant pressure and adaptation

### CLS level 1: adaptive team rules and visual cue processing

This warm-up activity helps players react to basic rule changes based on bib colors. Players are divided into colored teams, and the coach introduces simple rules during play. For example, White receives with the right foot, Green with the left, Blue with the chest. These instructions change without stopping the drill, requiring players to notice the cue and adjust their actions (Supplementary Figure S2A).

The goal is to encourage quick visual recognition and flexible response, while keeping technical and tactical demands low. As players get comfortable, more colors and quicker rule changes are introduced (Supplementary Figure S2B). For instance, the coach might say “Green cannot pass to Blue” or “Red now plays with White,” prompting players to reset their plan mid-action.

These adjustments build a foundation for cognitive adaptation and nonverbal coordination. This constant updating of behavior supports players’ ability to adapt to changing positional hierarchies in and out of possession without overwhelming players, as discussed earlier in this article. The drill fits CLS Level 1 due to its simplicity and use of well-known passing formats that allow for basic cognitive adaptation.

### CLS level 2: perception-based finishing with color-driven targets

This finishing drill integrates perceptual and decisional demands into a structured attacking pattern. The sequence begins with a pass from a wide player to a teammate making an overlapping run. At the moment of the overlap, the passer shouts a bib color (e.g., yellow), indicating the intended target inside the box (Supplementary Figure S3). The crosser must then scan, identify the player wearing that color, and deliver the ball accordingly.

This pass-overlap-color sequence adds a layer of attentional and visual processing. Players must respond to an external cue while staying in sync with movement and timing. Because the challenge is moderate and easily repeatable, this drill fits CLS Level 2, bridging technical execution with basic perceptual and decision elements.

### CLS level 3: role-based tactical drills with functional constraints

Cognitive constraints can also be embedded within full-team tactical drills by assigning bib colors to specific functional roles, for example, defenders in white, midfielders in red, and attackers in blue. Within this setup, the coach introduces interaction rules such as “White must play with Red” or “Red cannot pass to White,” which shape passing behavior without disrupting the structure of the formation. These constraints are introduced dynamically and can shift within the same drill (Supplementary Figure S4).

This structure allows coaches to manipulate the flow of play by targeting specific tactical behaviors. Rules can be used to encourage building from the back, create conditions for midfielders to receive facing forward, or simulate pressing by restricting passing options under pressure. Rather than prescribing fixed patterns, these drills force players to interpret evolving constraints, recognize their functional role, and adjust behavior accordingly.

Because these rules change dynamically and often under time pressure, players must rely on working memory, inhibition, and cognitive flexibility to make rapid, context-sensitive decisions. When applied in a full 11v11 format, the drill operates at CLS Level 3, due to the interaction between tactical structure and moderate-to-high cognitive demands. Simplified versions (such as 11v6 with predictable overloads), can reduce this demand and fall within lower CLS levels.

### CLS level 4: modified rondo with color and sequence-based constraints

Traditional rondos are widely used to train technical execution, quick decision-making, and spatial awareness. By integrating additional rule-based constraints, these drills can also target executive functions such as working memory, cognitive flexibility, and inhibition.

In one version, players must follow a passing sequence based on bib colors. For example, the coach might instruct: white → white → red, or set a rule such as “two passes to the same color, then change” (Supplementary Figure S5A). These constraints require players to hold sequences in mind while constantly updating spatial information, thereby increasing demands on working memory and attentional control. The challenge intensifies when touches are limited to one, requiring players to suppress automatic responses and make precise decisions under pressure.

Other variations ask players to memorize and reproduce longer sequences, or to reverse the order of passes when cued (Supplementary Figure S5B). These drills directly engage cognitive flexibility and response inhibition, skills shown to be relevant for decision-making under dynamic conditions (19, 20).

By embedding these cognitive demands into a dynamic and familiar format, this modified rondo fits CLS Level 4. It challenges players’ ability to track and apply evolving rules while under temporal and spatial constraints, supporting the development of game-relevant executive functions.

### CLS level 5: dual-task possession with conflicting objectives

This drill creates a high cognitive load by requiring players to manage two overlapping objectives simultaneously. Inside a square, two teams play a 4v4 possession game. Around them, four support players circulate a second ball. Players inside must keep possession while also tracking which outside player has the ball. When a condition is met (e.g., recognizing who has possession externally), they must pass a second ball to that player, disrupting the outer rotation (Supplementary Figure S6).

This activity challenges multiple cognitive processes. Players must constantly switch attention between internal play and external cues, a key executive function known as attentional shifting. They also need to inhibit impulsive actions (e.g., passing to the closest player) and instead follow the rule-dependent target. Working memory is taxed throughout, as players must hold task rules and spatial positions in mind while responding under time pressure.

Research in sport psychology shows that dual-task environments can impair decision speed and accuracy, especially under fatigue or uncertainty (21, 22). However, training under these conditions can improve resistance to cognitive overload and support better decision-making under pressure (23).

Given these demands, this drill aligns with CLS Level 5. It places players in a cognitively complex situation that closely reflects match demands during transitions, where rapid context switching and precise decisions are critical.

### Adapting CLS levels to age and skill context

Each CLS level is defined by core features (as shown in Table 1), but these should be viewed as adaptable frameworks rather than rigid templates. Age-related differences in cognitive development, particularly in working memory capacity and processing speed, should inform how drills are adjusted across different populations. For example, younger players typically show lower working memory span and slower cognitive processing than older adolescents and adults, which can affect their ability to respond to high cognitive load tasks (24–26). A Level 4 drill suitable for a senior team may require simplification for U12 players to ensure that the task remains challenging but not overwhelming.

Coaches should also consider the playing style of the club and individual differences in cognitive ability. Some players will naturally perform well under pressure and adapt quickly to cognitively demanding scenarios. For these athletes, specific constraints (such as requiring use of the non-dominant foot, limiting touches, or narrowing the space) can increase difficulty while keeping the overall drill structure intact. This flexibility allows the CLS to be both structured and scalable, promoting long-term development across age groups and skill levels.

### Player impressions following a season of CLS implementation

While the present article does not include formal experimental data, informal feedback from high-performance athletes aged 18 to 19 who trained under the CLS framework over the course of a year provides preliminary support for its perceived impact. These players were exposed to drills classified as levels 1 to 4 on the CLS and reported improved decision-making under pressure, as well as greater ease during matches compared to training intensity. As one player put it:

“In competition, I felt more confident in my decisions—they were quick and accurate. At the same time, my individual technique under pressure improved, because the training sessions during the week pushed me close to my limit. Many times, we felt that the exercises and training were harder than the match itself.” (M.D.)

Another commented:

“Matches became easier. During the week, the exercises forced us to think and act very quickly. My brain got used to making decisions under pressure.” (Á.N.)

A third player reflected on how the training aligned with collective tactical goals:

“I remember that year we wanted to keep possession for long periods. The training exercises made it easier for me to find the best-positioned teammate under pressure.” (T.M.)

Although anecdotal, this feedback highlights the potential of the CLS framework to influence players’ cognitive engagement and perceived performance. These observations may help guide future empirical validation and support the development of applied protocols.

## Tracking cognitive development in practice

While the CLS helps structure the cognitive demands of training, measuring how players respond to these demands is equally important. In applied settings, several tools can help coaches track cognitive engagement and player progression over time.

Video analysis, GPS data, and tagging platforms are particularly useful for observing how players adapt to these cognitive constraints. For example, in drills like *CLS Level 5: Dual-Task Possession with Conflicting Objectives*, analysts can observe how often players respond to all elements of the task or fall back on simpler strategies. This helps reveal whether players are actively processing the full set of constraints or avoiding cognitive load.

Another practical example can be seen in a defensive passing drill aimed at developing both technique and decision-making under time pressure (Supplementary Videos S1–S4). In this drill, a center-back must react to a color cue called out by the coach and immediately deliver a pass to a moving target that matches the cue. As the drill progresses, complexity increases across four phases: (1) a basic version with static options, (2) added cue-based decisions, (3) moving targets that increase difficulty, and (4) the coach pressing the player right after the cue to simulate match-like pressure and reduce processing time.

By recording the session on video, analysts can tag individual trials to measure: reaction time, pass accuracy, and decision success across repetitions. Over time, this provides valuable insight into the player’s cognitive development with practice. These behavioral markers can be used to individualize progression, compare players within a group, or identify cognitive profiles that may benefit from targeted interventions.

In addition to performance metrics, subjective assessments like the NASA-TLX can give insight into how players perceive the cognitive demands. Combined, these tools create a practical way to monitor whether the CLS is effectively challenging and developing key mental processes during training.

## Contextualizing the CLS within cognitive and perceptual training approaches

To situate the Cognitive Load Spectrum (CLS) within the broader landscape of applied sport science, it is important to engage with established perceptual-cognitive training models that have influenced both theory and practice. One of the most prominent is Vickers’ Quiet Eye theory, which highlights the role of sustained visual fixation during motor execution, suggesting that expert performers stabilize gaze to optimize planning and reduce performance variability (27). Another influential approach involves dual-task paradigms, where athletes perform motor tasks while concurrently managing additional cognitive demands. These studies have shown how attentional resources and working memory interact with motor performance under pressure, offering valuable insights into expertise and decision-making in sport (28, 29).

The CLS sits alongside these established frameworks as a complementary tool that expands the possibilities for applying cognitive principles in sport training. While models like Quiet Eye and dual-task paradigms offer powerful insights into specific mechanisms such as attentional control and multitasking, the CLS contributes by providing a broader structure to intentionally vary cognitive demands across multiple domains. Rather than replacing or contrasting with these approaches, the CLS can help integrate different types of cognitive challenges within a coherent progression, supporting athlete development in a way that reflects the demands of the game and the range of cognitive skills required.

Recent work applying cognitive load theory in other team sports (e.g., basketball and hockey) has shown how manipulating the complexity of training tasks can influence tactical decision-making and cognitive adaptation under pressure (30, 31). These studies support the broader relevance of load-based frameworks like the CLS and point to opportunities for interdisciplinary collaboration across applied sport contexts.

## Conclusion

In this article, we proposed that spatial intelligence can be trained systematically through ecologically valid drills when cognitive constraints are carefully designed. By applying the CLS, coaches can classify and adapt practices based on the demands they place on perception, memory, and decision-making, without detaching from the natural flow of the game.

This framework supports a shift from reactive coaching to intentional cognitive design. By embedding memory load, attentional shifts, and constraint-based variability into training, players engage with the same types of demands they navigate during matches. Rather than separating cognitive training from football, here, we propose a bridge, transforming on-pitch routines into environments to enhance players’ cognitive abilities.

Collaborative research between neuroscientists and football clubs may play a central role in advancing this approach. Neuroscientists can help identify the cognitive traits embedded in existing drills, refine them to increase cognitive challenge, and assess how these traits relate to performance. The CLS offers a shared language to guide this interaction. In addition, neuroscientific tools can support the analysis of how players engage spatial working memory during high-CLS sessions by linking on-pitch decisions to internal memory processes. For instance, decision-making patterns inferred from player positioning and action options, extracted via GPS or video-based tracking, can reveal the cognitive processes faced during training. These analyses may help quantify a player’s cognitive profile within their age group or positional role. If an athlete consistently shows lower spatial working memory engagement, this could also inform off-pitch interventions aimed at improving focus, or cognitive load.

Finally, implementing cognitively demanding training, especially in youth populations, requires careful ethical consideration. Coaches should be attentive to signs of cognitive fatigue and adjust sessions accordingly. In this context, using training periodization can help distribute cognitive load more effectively over time, balancing stimulation with recovery. Additionally, age-appropriate scaling of cognitive difficulty and avoiding excessive or prolonged exposure to high mental demands are important to ensure that training remains beneficial, safe, and developmentally appropriate.

## Data Availability

The original contributions presented in the study are included in the article/supplementary material, further inquiries can be directed to the corresponding author.
